# AI Revolution in Radiology, Radiation Oncology and Nuclear Medicine: Transforming and Innovating the Radiological Sciences

**DOI:** 10.1111/1754-9485.13880

**Published:** 2025-07-09

**Authors:** S. Carriero, R. Cannella, F. Cicchetti, A. Angileri, A. Bruno, P. Biondetti, R. R. Colciago, A. D'Antonio, G. Della Pepa, F. Grassi, V. Granata, C. Lanza, S. Santicchia, A. Miceli, A. Piras, V. Salvestrini, G. Santo, F. Pesapane, A. Barile, G. Carrafiello, A. Giovagnoni

**Affiliations:** ^1^ Department of Diagnostic and Interventional Radiology Foundation IRCCS Cà Granda‐Ospedale Maggiore Policlinico Milan Italy; ^2^ Department of Biomedicine, Neuroscience and Advanced Diagnostics (BiND) University of Palermo Palermo Italy; ^3^ Postgraduate School of Diagnostic and Interventional Radiology University of Milan Milan Italy; ^4^ Department of Clinical, Special and Dental Sciences University Politecnica Delle Marche Ancona Italy; ^5^ School of Medicine and Surgery University of Milan Bicocca Milan Italy; ^6^ Department of Advanced Biomedical Sciences University of Naples “Federico II” Napoli Italy; ^7^ Breast Radiology Unit Fondazione IRCCS Istituto Nazionale Dei Tumori Milano Italy; ^8^ Division of Radiology Università degli Studi della Campania “Luigi Vanvitelli” Naples Italy; ^9^ Istituto Nazionale Tumori, IRCCS, Fondazione Pascale Napoli Italy; ^10^ Nuclear Medicine Unit Azienda Ospedaliero‐Universitaria SS. Antonio e Biagio e Cesare Arrigo Alessandria Italy; ^11^ UO Radioterapia Oncologica Palermo Italy; ^12^ RI.MED Foundation Palermo Italy; ^13^ Department of Health Promotion, Mother and Child Care, Internal Medicine and Medical Specialties, Molecular and Clinical Medicine University of Palermo Palermo Italy; ^14^ Radiation Oncology Mater Olbia Hospital Olbia Italy; ^15^ Radiation Oncology Unit, Oncology Department Azienda Ospedaliero Universitaria Careggi Florence Italy; ^16^ Nuclear Medicine Unit, Department of Experimental and Clinical Medicine “Magna Graecia” University of Catanzaro Catanzaro Italy; ^17^ Breast Imaging Division IEO European Institute of Oncology IRCCS Milan Italy; ^18^ Department of Biotechnology and Applied Clinical Sciences University of L'Aquila L'Aquila Italy; ^19^ Department of Radiology Ospedali Riuniti, Università Politecnica Delle Marche Ancona Italy

**Keywords:** artificial intelligence, deep learning, machine learning, nuclear medicine, radiation oncology, radiology, resident

## Abstract

The integration of artificial intelligence (AI) into clinical practice, particularly within radiology, nuclear medicine and radiation oncology, is transforming diagnostic and therapeutic processes. AI‐driven tools, especially in deep learning and machine learning, have shown remarkable potential in enhancing image recognition, analysis and decision‐making. This technological advancement allows for the automation of routine tasks, improved diagnostic accuracy, and the reduction of human error, leading to more efficient workflows. Moreover, the successful implementation of AI in healthcare requires comprehensive education and training for young clinicians, with a pressing need to incorporate AI into residency programmes, ensuring that future specialists are equipped with traditional skills and a deep understanding of AI technologies and their clinical applications. This includes knowledge of software, data analysis, imaging informatics and ethical considerations surrounding AI use in medicine. By fostering interdisciplinary integration and emphasising AI education, healthcare professionals can fully harness AI's potential to improve patient outcomes and advance the field of medical imaging and therapy. This review aims to evaluate how AI influences radiology, nuclear medicine and radiation oncology, while highlighting the necessity for specialised AI training in medical education to ensure its successful clinical integration.

**Abbreviations**
AI: artificial intelligence
DL: deep learning
ML: machine learning
NM: nuclear medicine
RO: radiation oncology
RT: radiation therapy

## 1 Introduction

Artificial intelligence (AI) integration in the radiological area has emerged as a transformative force, promising to reshape the landscape of diagnostic imaging and radiation oncology (RO). Recent advancements in AI, particularly in deep learning (DL) and machine learning (ML) algorithms, have significantly enhanced image recognition, interpretation and decision‐making processes [1, 2, 3, 4]. This is a rapid transformation from a discipline based on the visual interpretation of the images toward a new type of imaging, which integrates the quantitative data (biomarkers) coming from the images with the interpretative modality, since the extracted quantitative features can be analysed to reflect the underlying pathophysiology [5, 6]. As a result, AI is increasingly being recognised not just as a complementary tool but as a critical component in the clinical workflow [7].

The impetus for this review stems from a recent meeting at the first joint congress, the so‐called ‘Next Generation Congress’, of the Italian radiological area held in Milan in June 2024, which brought together young residents and specialists in radiology, radiation oncology and nuclear medicine (NM). This gathering underscored the growing relevance of AI in clinical practice and highlighted the urgent need for its widespread dissemination and adoption, particularly among the next generation of practitioners [8, 9, 10].

The discussions emphasised that, while AI has already begun to make significant inroads into the field, its full potential remains largely untapped, particularly in routine clinical settings. In current diagnostic and therapeutic clinical practice, AI's most notable contributions include the automation of image analysis, enhancement of diagnostic accuracy and reduction of human error [11]. For instance, AI algorithms have demonstrated superior performance in detecting pathologies such as pulmonary nodules, breast lesions and intracranial haemorrhages, often with speed and precision surpassing human radiologists [12]. Furthermore, AI has shown potential to improve workflow efficiency by automating routine tasks such as image sorting, triage, and even preliminary reporting of some diagnostic examinations, allowing radiologists to focus on more complex cases [13, 14, 15].

Despite these advances, the adoption of AI in daily clinical practice is challenging. There is an ongoing debate about the ethical implications of AI, particularly concerning data privacy, the potential for algorithmic bias, and the overarching question of whether AI might one day supplant the physician [16]. However, the consensus among experts is that AI is more likely to augment rather than replace human expertise, serving as a powerful adjunct in the diagnostic process [17, 18, 19].

A critical aspect of this transition is educating and training young residents and specialists in the radiological area. The next generation must be equipped not only with traditional skills but also with a deep understanding of AI technologies and their applications. This requires an updated curriculum integrating AI into radiology, RO and NM training programmes, fostering a generation of specialists adept at using these tools to enhance patient care [20, 21, 22].

This review aims to provide an overview of the current state of AI in radiology, RO and NM, highlight its practical applications, and discuss strategies for fostering AI adoption among specialists.

## 2 The Importance of Teaching AI During Residency

Before exploring the specific reasons why AI should be taught to radiology, NM and RO residents, it is essential to understand the key advantages of AI in medical education. The development of Generative Artificial Intelligence (GenAI) models, such as Large Language Models (LLMs), is creating a disruption in educational landscapes. GenAI offers significant opportunities to personalise educational programmes, automate routine tasks, create tailored content, and enhance access to evidence‐based information that could fill the gaps in resident clinical preparation, hypothetically leading to a reduction in the time needed to prepare the trainees adequately [23, 24]. However, the integration of GenAI into medical education raises critical challenges and concerns about data privacy, accuracy, and the risk of over‐reliance on AI, highlighting the importance of equipping the younger generation of clinicians, particularly radiologists, nuclear medicine physicians and radiation oncologists, with a robust understanding of AI's strengths and limitations [24]. Addressing these issues and preparing future healthcare professionals to navigate AI's potential and risks will be essential for the responsible and effective implementation of AI in healthcare.

## 3 The Importance of Teaching AI to Young Radiologists

The rapid spread of AI in clinical practice creates an urgent need to improve the education and training of young radiologists to fully understand the potential and advantages of AI applications in patient clinical care [25, 26, 27]. Implementing AI by young radiologists requires basic knowledge of software use, data analysis and imaging informatics fundamentals, data safety, potential biases, ethical considerations and evolving regulations of AI [22, 28, 29]. In addition, the necessity of tailored educational programmes is an emerging demand among young radiologists [30, 31]. Most young radiologists and trainees believe that AI basics should be taught during residency, but the actual implementation in residency programmes remains scant [32, 33]. In a multi‐institutional survey, 83% of residents in the United States agree that AI education should be part of the radiology residency curriculum [34]. Similarly, in an international survey, 79% of radiologists and radiology residents reported that AI should be incorporated into residency programmes, and lack of knowledge was considered a hurdle for AI implementation by 57% of the responders [35]. In a survey among members of the SIRM Lombardy, young radiologists under the age of 30 considered themselves not adequately informed on the AI topics, with significant difference of opinion compared to older radiologists [36].

Most of the current education on AI in radiology is based on conference meetings, scientific literature, workshops by commercial companies, and dedicated online training. Particularly, commercial companies worldwide offer a large variety of AI theoretical or hands‐on courses to improve young radiologists' knowledge and skills, while the involvement of academic institutions is still limited [37, 38]. More structured training should be provided during the radiology residency to ensure an impartial and industry‐unbiased education of young radiologists [39, 40, 41]. In this context, the training curriculum of the European Society of Radiology recommends teaching basic principles of medical imaging informatics and AI tools during general radiology training (level I–II) and learning knowledge of clinical applications of AI during subspecialization in radiology (level III) [42].

## 4 The Importance of Teaching AI to Young Nuclear Medicine Physicians

Much like radiology, NM has experienced a rapid evolution with the integration of AI into clinical practice. This advancement encompasses improvements in instrumentation, image acquisition and formation, image reconstruction, low‐dose and fast scanning techniques, imaging quantification, image interpretation—including computer‐aided detection, diagnosis and prognosis—as well as internal radiation dosimetry for radioligand therapy [43, 44, 45, 46, 47, 48, 49].

The convergence of AI and NM has created a synergistic relationship that promises groundbreaking opportunities for the future. However, to fully realise this potential, overcome current limitations and keep pace with the swift progression of AI technologies, it is essential to foster a controlled cultural shift toward innovation through comprehensive training and education [10, 50]. Disseminating knowledge about implementing AI in NM and molecular imaging is greatly needed, and educating young physicians in this area will be crucial for the sustained advancement of the discipline.

Effective implementation of AI must be accompanied by robust educational initiatives to ensure that no one is left behind. This requires a thorough understanding of software, data analysis, imaging informatics, data safety, potential pitfalls, ethical considerations, and the evolving regulations surrounding AI [50, 51]. In this context, a significant effort should be made by academia to adapt the training programme to this evolving scenario by organising dedicated courses involving experts in the field who can adequately teach and train students and physicians in this new avenue of NM. Developing educational programmes within NM training schools that address scientific and clinical aspects will help current and future scientists and clinicians become familiar with and adept at using AI, enhancing its adoption and development.

The European School of Multimodality Imaging and Therapy (ESMIT), the educational branch of the European Association of Nuclear Medicine (EANM), has already initiated efforts in this direction by offering webinars and advanced courses at the EANM Educational Facility in Vienna, such as the 2023 course titled ‘Artificial Intelligence in Imaging: Making the Future’ [45]. This starting commitment to education will ensure that NM remains at the forefront of medical innovation, well‐prepared to meet the challenges and seize the opportunities presented by AI advancements [52].

## 5 The Importance of Teaching AI to Young Radiation Oncologists

AI is revolutionising many fields of medicine, including RO, where precision and efficiency are critical aspects [53, 54, 55, 56]. AI can assist in processing medical images and planning treatments, allowing for more accurate identification and localisation of tumours. This helps personalise therapies, minimise damage to healthy tissues, and increase the treatment's effectiveness [57, 58, 59].

The impact of AI on radiation therapy (RT) is significant, with the potential for AI and ML to excel in various aspects of the RT process. These technologies not only improve the quality of training for residents but are also becoming an increasingly essential resource in the medical field [60, 61].

A recent systematic review by the Young Group of the Italian Association of Radiotherapy and Clinical Oncology (yAIRO) highlighted how AI and radiomics seem to significantly impact image‐guided radiation therapy (IGRT) in all the phases of RT workflow [62]. Young radiation oncologists expressed both enthusiasm and concern about automatic segmentation, as revealed by a survey conducted by the SFjRO (Société Française des jeunes Radiothérapeutes Oncologues) [63]. Many participants supported incorporating these tools into training to ensure future radio‐oncologists maintain a strong knowledge of radio‐anatomy. However, one‐third of respondents suggested restricting AI tools to senior physicians to prevent neglect of this essential knowledge [63].

Guckenberger et al. evaluated the potential of the AI chatbot ChatGPT in supporting young clinical scientists. ChatGPT was generally favoured by less experienced researchers, but it did not bridge the gap in scientific expertise; in fact, less experienced researchers were more likely to accept the false results produced uncritically by the AI [64].

Training young radiation oncologists on AI is a technical necessity and an essential step to ensure high‐quality, safe and leading‐edge medicine. Teaching AI to young radiation oncologists is crucial for several reasons, including improving the quality of care, process optimisation, innovation and research, and preparing for the future [60, 65, 66, 67].

## 6 AI: Clinical Application

### 6.1 AI in Radiology: Clinical Application

AI is playing an increasingly transformative role in radiology. To date, a total of 213 AI‐based software applications have received CE approval, which is a European certification indicating conformity with health, safety and performance standards; hundreds more are currently under development and validation. The latest comprehensive list of these approved AI tools is available in the official registry, accessible at this link: https://radiology.healthairegister.com [68, 69, 70].

AI algorithms primarily target neuroradiology and chest radiology, followed by breast, abdominal and musculoskeletal imaging applications [71, 72, 73, 74, 75, 76, 77, 78]. AI applications span across all radiological modalities, including radiography, computed tomography (CT), magnetic resonance (MR) and ultrasound (US) [79, 80, 81, 82, 83], and can be classified into two categories: interpretative and non‐interpretative uses [68, 84]. Broadly speaking, interpretative uses encompass the processes of detection, characterisation and monitoring. Of these, detection applications were the first to be developed, and they remain the most widely validated and implemented AI tools in clinical practice [85].

**FIGURE 1:**
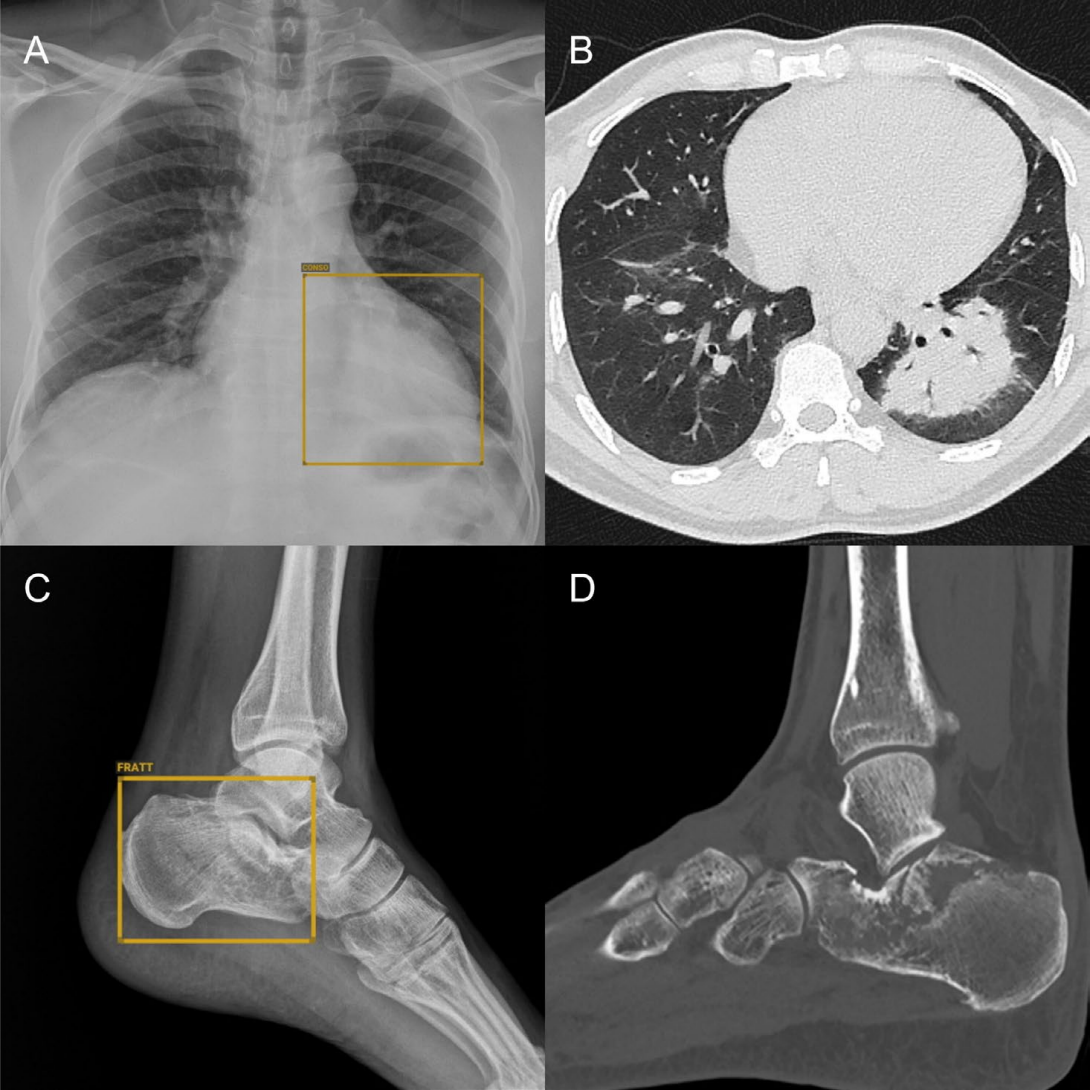
Examples of AI‐assisted diagnosis in radiology. Chest radiograph (A) showing a subtle retrocardiac lung consolidation identified by AI (ChestView, Gleamer), and confirmed on CT (B). Bone radiograph (C) acquired in a trauma patient with calcaneus fracture detected by AI (BoneView, Gleamer) and confirmed on CT (D).

When examining the areas where AI has advanced the most, neuroradiology stands out, with key applications centred around optimising stroke workflows, detecting large vessel occlusions, quantifying stroke scoring metrics, segmenting and assessing ischaemic or at‐risk tissue, and predicting clinical outcomes in stroke patients [86]. In chest radiology, the most prominent applications are in the field of lung cancer, focusing on lesion detection, segmentation, and characterisation [72, 87], as well as in the emergency setting (Figure 1) [88, 89]. In breast radiology, the first area where AI was extensively studied and has achieved the widest global adoption is mammographic screening, which involves lesion detection [90, 91]. Building on the success in these domains, AI applications in breast are now being tested across all imaging modalities, with an increasingly prominent role in lesion characterisation [90, 92, 93]. A list of AI applications across the main fields of radiology is provided in Table 1.

**TABLE 1:** A list of AI applications across the main fields of radiology.

Subspecialty	Main applications [94]
Neuroradiology	Detection of strokes, haemorrhages and aneurysms, alongside the assessment and segmentation of brain structures
Chest radiology	Detection of lung nodules, pneumonia, pneumothorax and rib fractures. It also includes the analysis of lung diseases and emphysema quantification
Breast radiology	Lesion detection and characterisation, breast density analysis
Abdominal radiology	Liver segmentation, genitourinary structure analysis, lesion identification, characterisation of focal liver lesions and adrenal lesion characterisation
Musculoskeletal radiology	Detection of fractures and osteoarthritis, and the quantitative imaging of bone structures
Cardiovascular radiology	Coronary artery assessments, myocardial infarction diagnostics and evaluation of cardiomyopathies alongside general cardiac function
Emergency radiology	Rapid identification of haemorrhages, occlusions, fractures and various abdominal emergencies

Non‐interpretative uses of AI include tools designed to enhance workflow efficiency, support clinical protocols, optimise scanner and patient scheduling, and facilitate structured radiological reporting through language processing [84, 95, 96]. AI is becoming increasingly integrated into both clinical practice and research. As asserted by Langlotz [97], while it is unlikely that radiologists will be replaced by AI, those who do not adopt AI will undoubtedly be outpaced by those who do. This principle underpins scientific progress across all fields, particularly in healthcare. It also highlights the importance of understanding the foundational principles and ethical considerations necessary for the full and responsible implementation of AI in radiology [98, 99, 100].

### 6.2 AI in Nuclear Medicine: Clinical Application

As mentioned above, the development of AI represents a breakthrough in NM, providing many applications. Besides the widely spread AI‐driven managing pipelines, including patient scheduling, report generation and images management, the main clinical applications of AI in NM involve images generation, post‐processing and analysis [45, 101, 102].

Recently, DL and ML models were trained and implemented both in the traditional iterative reconstruction process and post‐processing analysis, optimising image quality with significant dose reduction, shortening acquisition time, and extracting imaging biomarkers that may evaluate clinical endpoints. With the spreading of hybrid imaging (PET/CT, PET/MR and SPECT/CT), many studies aimed to develop AI‐assisted tools for image attenuation and motion correction, with promising results [43, 46, 103, 104].

**FIGURE 2:**
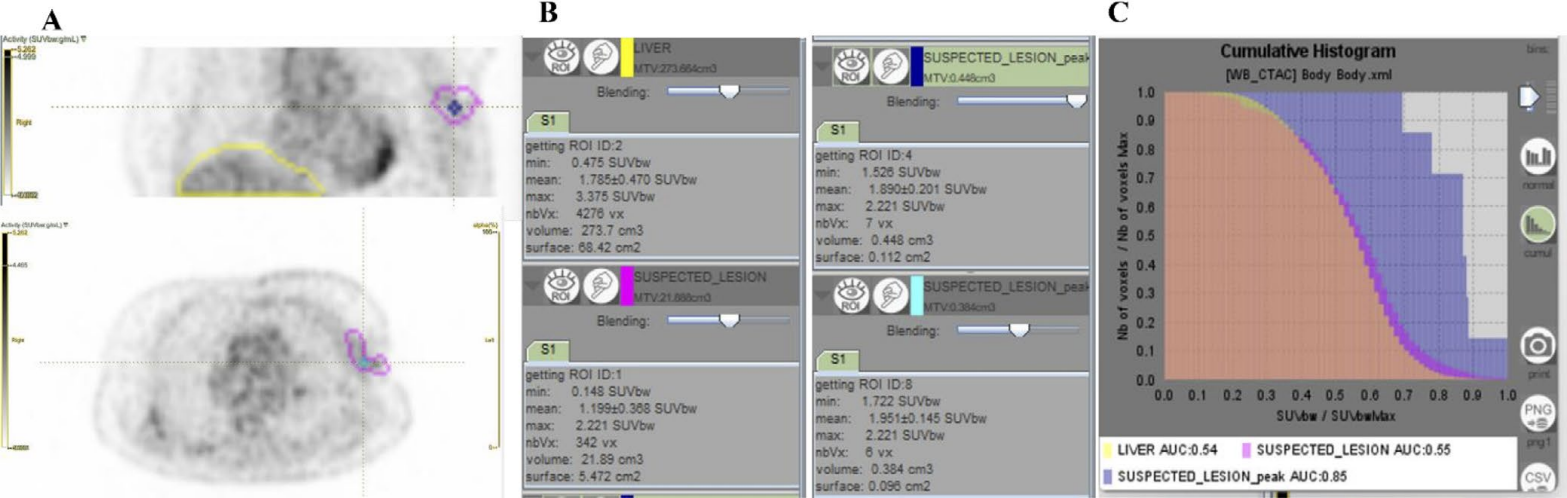
AI‐assisted interpretation of an ^18^F‐FDG PET‐CT scan. Standard PET images (A) highlight a suspicious area of uptake in the left axilla (pink), with the maximum uptake peak marked (blue circle) and liver uptake shown for reference (yellow). The AI algorithm automatically segmented the regions and quantified uptake values in both the suspicious area and the liver, ultimately suggesting a benign nature (B). Cumulative histograms illustrate the voxel‐wise activity distribution across all analysed regions (C). In the specific case, the lesion was initially equivocal, with differential diagnosis including metastatic recurrence of infiltrating ductal carcinoma (pT2N1) versus post‐radiotherapy inflammatory changes. Histological examination confirmed granulomatous tissue, consistent with the benign assessment provided by the AI.

Currently, the main improvement gained by AI analysis involves image segmentation, especially in oncologic, cardiac and neurologic NM imaging [103, 104]. In oncologic imaging, the feasibility of AI‐assisted image analysis for detecting malignant lesions, automating segmentation, and extracting radiomic features—which may predict disease progression and therapy response—can also estimate whole‐body dosimetry in patients referred for radioligand therapy [47, 104] (Figure 2).

The growing use of target‐specific PET tracers (e.g., ^18^F‐PSMA‐1007 and ^68^Ga‐PSMA in prostate cancer, ^18^F‐FES in breast cancer) allows the development of further algorithms predicting histologic diagnosis and molecular tumour classification [105, 106, 107, 108, 109].

Myocardial perfusion imaging (MPI) by SPECT or PET is another broad field of AI application [110]. AI allows nuclear cardiology studies to be acquired with lower doses or shorter acquisition times, also providing synthetic attenuation correction of SPECT images to improve diagnostic accuracy [111, 112]. ML and AI‐assisted algorithms also showed a good performance in myocardial segmentation compared to experienced NM physicians. Moreover, ML‐extracted quantitative features have good prognostic value in detecting obstructive coronary disease and predicting cardiac adverse events when integrated with clinical data [112, 113, 114].

Technical innovation in AI has led to considerable advances in brain imaging as well. Along with the already proven usefulness in image quality assessment and segmentation, many authors demonstrated the added value of DL methods for image analysis in identifying neurodegenerative disorders and predicting disease evolution (e.g., ^123^I‐Ioflupane in Parkinson's disease, ^18^F‐FDG and ^18^F‐Florbetaben in Alzheimer's disease) [115, 116, 117, 118, 119, 120].

AI applications may, therefore, help enhance the role of NM in several clinical scenarios, with promising results. AI enables patient‐tailored management in various oncologic, cardiologic and neurological settings. However, further and wider studies are needed to standardise the different algorithms and obtain reliable parameters from different DL models.

### 6.3 AI in Radiation Oncology: Clinical Application

RO is closely tied to technological advancements, and the emergence of AI has the potential to revolutionise this field. AI can accelerate processes, improve outcomes, and reduce the workload in daily clinical practice, as shown in Table 2 [54, 121, 122, 123]. Xiao et al. used a DL‐based tool to enhance the segmentation of organs at risk in cervical cancer [124]. In a study of 313 patients, automatic contouring was compared to manual segmentation by expert radiation oncologists. The AI achieved a Dice similarity coefficient between 0.91 and 0.98 for most organs, with an average segmentation time of 6.6 s. The rectum had the poorest performance, likely due to its small size and unclear boundaries [124]. Similarly, Liao et al. developed a DL tool for segmenting 16 organs at risk in the abdomen using 544 CT scans. The tool provided accurate contouring for the liver, kidneys and spleen, with minor errors in organs like the stomach, oesophagus, adrenal glands and rectum. Major revisions were needed only for the colon and small bowel contours [125].

**TABLE 2:** How artificial intelligence improves radiation oncology.

Function	AI contribution
Contouring	AI provides accurate contouring in a very small time
Planning	AI improves dose distribution to OARs while maintaining dose coverage of the target
Adapting	AI quantifies structural changes, achieving good adaptation to anatomic changes
Predicting	AI predicts clinical responses, leading to more tailored treatment

In the field of non‐small‐cell lung cancer, Hosny et al. conducted an observational study to validate DL models for target segmentation. While the model performed well in internal validation, it did not surpass the benchmark when tested with external data from different experts. However, AI assistance led to a significant 65% reduction in segmentation time and a 32% reduction in inter‐observer variability [126]. Zeverino et al. reported the successful clinical implementation of an auto‐planning tool for left‐sided breast cancer [127]. The AI system planned 17 clinical treatments, which were then compared to manual plans. Compared to manual planning, the AI achieved less than 1% error and improved the dose distribution to organs at risk, such as the lungs and the left anterior descending artery [127]. During RT, Lee et al. developed a DL algorithm to predict weekly anatomical changes in lung tumours and the oesophagus. This tool accurately predicted structural changes, resulting in a 4.2 Gy reduction in the mean oesophageal dose while maintaining adequate tumour coverage [128].

Finally, AI is also proving valuable as a predictive tool. Li et al. created a DL approach to predict clinical outcomes based on magnetic resonance imaging. Applied to 99 rectal cancer patients, this AI model predicted a complete clinical response with an accuracy of 0.789, a specificity of 0.725, and a sensitivity of 0.812 [129].

## 7 AI: Unresolved Issues and Emerging Challenges

As AI progresses, its use in healthcare, especially in all radiological fields, requires careful ethical and legal evaluation. To accomplish this goal, the European Union published the so‐called ‘AI Act’ in 2024. The act classifies AI applications into four risk levels, with medical use designated as High‐Risk [130]. This category includes AI systems that may pose significant threats to health, safety, or fundamental rights, necessitating strict adherence to standards of quality, transparency, human oversight and safety [130].

Another essential aspect that must be considered to properly understand AI functionality is knowing that AI in healthcare presents specific risks, including algorithmic bias, errors and issues, such as AI hallucinations, where models generate incorrect but plausible results [131]. Furthermore, an article by Gichoya et al. explains that bias in medical imaging AI can emerge throughout the entire AI lifecycle, from design to deployment, underscoring the need to create and use diverse and representative datasets, develop and test rigorous validation protocols, and perform ongoing monitoring and evaluation of model performance to minimise these biases [132]. These measures are essential to promote fair and equitable healthcare outcomes through responsible AI development and implementation. Mitigating these risks requires technical solutions and a collaborative approach, engaging clinicians, AI developers, and ethicists to build trustworthy AI systems that enhance rather than undermine the quality of healthcare delivery.

## 8 Conclusions and Perspectives

The integration of AI into clinical practice presents a tremendous opportunity to enhance diagnostic accuracy and operational efficiency, particularly in disciplines such as radiology, NM and RO. The success of this transformation depends on the collaboration and integration of these fields, as AI enables more comprehensive and precise approaches to patient care. By combining the strengths of each specialty, AI facilitates the development of Precision Medicine, which should no longer be an unattainable utopia but a realistic and achievable goal [133]. This integration allows for tailored diagnostic and therapeutic strategies, improving outcomes by addressing each patient's unique needs.

A critical component of ensuring this evolution is education. Future physicians must be trained not only in traditional clinical skills but also in the use and understanding of AI technologies. Teaching AI during the training is no longer optional but necessary to equip them to navigate an ever‐evolving field. Only through updated and continuous educational programmes can the widespread and safe adoption of AI technologies be guaranteed, enabling next generation professionals to improve patient care and contribute to the advancement of modern medicine.

## Conflicts of Interest

The authors declare no conflicts of interest.

## Data Availability

The authors have nothing to report.
